# Quantifying biomass consumption and carbon release from the California Rim fire by integrating airborne LiDAR and Landsat OLI data

**DOI:** 10.1002/2015JG003315

**Published:** 2017-02-18

**Authors:** Mariano Garcia, Sassan Saatchi, Angeles Casas, Alexander Koltunov, Susan Ustin, Carlos Ramirez, Jorge Garcia‐Gutierrez, Heiko Balzter

**Affiliations:** ^1^ Jet Propulsion LaboratoryCalifornia Institute of TechnologyPasadenaCaliforniaUSA; ^2^ Centre for Landscape and Climate ResearchUniversity of LeicesterLeicesterUK; ^3^ Center for Spatial Technologies and Remote SensingUniversity of CaliforniaDavisCaliforniaUSA; ^4^ Region 5 Remote Sensing Lab, McClellanUSDA Forest ServiceVallejoCaliforniaUSA; ^5^ Department of Computer ScienceUniversity of SevilleSevilleSpain; ^6^ National Centre for Earth ObservationUniversity of LeicesterLeicesterUK

**Keywords:** biomass consumption, carbon emissions, megafires, lidar, Landsat OLI, data integration

## Abstract

Quantifying biomass consumption and carbon release is critical to understanding the role of fires in the carbon cycle and air quality. We present a methodology to estimate the biomass consumed and the carbon released by the California Rim fire by integrating postfire airborne LiDAR and multitemporal Landsat Operational Land Imager (OLI) imagery. First, a support vector regression (SVR) model was trained to estimate the aboveground biomass (AGB) from LiDAR‐derived metrics over the unburned area. The selected model estimated AGB with an *R* ^2^ of 0.82 and RMSE of 59.98 Mg/ha. Second, LiDAR‐based biomass estimates were extrapolated to the entire area before and after the fire, using Landsat OLI reflectance bands, Normalized Difference Infrared Index, and the elevation derived from LiDAR data. The extrapolation was performed using SVR models that resulted in *R* ^2^ of 0.73 and 0.79 and RMSE of 87.18 (Mg/ha) and 75.43 (Mg/ha) for the postfire and prefire images, respectively. After removing bias from the AGB extrapolations using a linear relationship between estimated and observed values, we estimated the biomass consumption from postfire LiDAR and prefire Landsat maps to be 6.58 ± 0.03 Tg (10^12^ g), which translate into 12.06 ± 0.06 Tg CO2_e_ released to the atmosphere, equivalent to the annual emissions of 2.57 million cars.

## Key Points

- Quantification of biomass consumption and carbon release by a megafire
- Integration of airborne LiDAR and Landsat OLI data to estimate biomass consumption
- Uncertainty of estimates calculated and provided for each biomass consumption estimation

## 1 Introduction

Forest fires are a natural process playing a critical role in the structure and functioning of many terrestrial ecosystems that are adapted to fires. They are a key element of forest composition and succession, resulting in stand thinning, removing understory vegetation, and regulating patterns of carbon (C) accumulation by promoting vertical stratification of the forest canopy [*Chuvieco*, 2008; *French et al*., 2003]. Whereas low to moderate severity fires may increase nutrients and promote vegetation renovation, high burn severity fires reduce organic matter and deteriorate soil structure and porosity [*Certini*, 2005; *Neary et al*., 1999]. Forest fires also impact the C cycle through the direct release of C into the atmosphere during the biomass burning [*Andreae*, 1991; *French et al*., 2003; *van der Werf et al*., 2006]. Therefore, quantifying the impact of fires on ecosystem structure and C emissions from biomass burning are important elements of evaluating the interactions of climate and ecosystems.

Climate condition is a key driver of fire occurrence. In areas where climate change leads to warmer and drier future conditions, the frequency and the intensity of fires are expected to increase, causing significantly larger impacts on ecosystem function and its role on the global carbon cycle [*Liu et al*., 2013; *Stocks et al*., 1998]. In addition, areas with increased fuel load, due to the loss of traditional woodcutting activities and fire exclusion policies, may adversely favor the occurrence of extreme fires with large socioeconomic and ecological consequences. These so‐called megafires [*Attiwill and Binkley*, 2013; *San‐Miguel‐Ayanz et al*., 2013] release to the atmosphere huge amounts of C accumulated during decades of tree growth. The expected increase in the intensity and frequency of megafires [*Attiwill and Binkley*, 2013] requires improved accounting of the C released by these fires and better characterization of their role in the climate system.

Quantification of the C emissions from biomass burning can be achieved using remote sensing by either direct top‐down measurement of trace gases released during the fire [*Arellano et al*., 2004; *Kaufman et al*., 1992] or by an indirect bottom‐up modeling approach in which burned area observations are combined with ecosystem biomass and fuel data [*Seiler and Crutzen*, 1980]

(1) $$C_{r}=A\times B\times f_{c}\times \beta \text{,}$$

where *A* is the area burned (in hectare), *B* is the biomass density (Mg ha^−1^), *f_c_* is the fraction of the biomass that is carbon, and *β* is the combustion completeness (CC) or burning efficiency, i.e., the fraction of biomass consumed during burning.

Uncertainties in the C release derived from the model by *Seiler and Crutzen* [1980] result from the uncertainty in each of its parameters. *van der Werf et al*. [2006] pointed out *A* as the most uncertain parameter at a global scale; however, uncertainties in biomass density and combustion completeness significantly impact emission estimates as well. Fuel load, i.e., the amount of biomass available for burning, is usually estimated based on biome‐averaged values, which have large uncertainties due to the spatial variability in biomass within the biome [*van der Werf et al*., 2006; *van Leeuwen et al*., 2014] and may not represent well the biomass density of the affected area. More recently, biogeochemical models that account for the effect of herbivory and fuel wood collection have been used to estimate fuel load [*van der Werf et al*., 2006, 2010]. Nevertheless, the accuracy of the fuel load estimates is subject to the difficulty of parameterizing the model appropriately.

Thus, development of more accurate methods for estimating aboveground biomass and the fraction of the biomass consumed during the fire is crucial to improve and validate fire emission estimates, as well as to correct estimates of fuel loads yielded by biogeochemical models used at regional and global scales.

Remote sensing provides a robust way of deriving fuel load. In particular, airborne and satellite LiDAR measurements have allowed development of novel techniques to accurately quantify the vertical and horizontal structures and the aboveground biomass over a wide variety of forest ecosystems [*García et al*., 2012; *Hudak et al*., 2012; *Næsset et al*., 2013]. As most LiDAR measurements are currently acquired from airborne platforms, the data are often limited in terms of spatial and temporal coverage. This hinders the analysis of prefire and postfire ecosystem status, in most cases due to the lack of data that would enable the characterization of aboveground biomass before the fire. On the other hand, multispectral optical and Radar sensors onboard satellites provide wide‐area coverage and frequent observations, which makes these sensors particularly well suited for monitoring fuel load dynamics. As a result, integration of airborne LiDAR with optical or Radar satellite observations provides a convenient alternative to overcome the shortcomings of LiDAR data availability, allowing for more accurate representation of the fuel load and the biomass dynamics, thus improving fire emission estimates.

Here we explore both the spatial and temporal extrapolation of the LiDAR data with multispectral Landsat OLI (Operational Land Imager) data over large areas to quantify the biomass before and after the fire. We also quantify the burned fuel consumption and the amount of carbon released during the Rim megafire in California. The LiDAR data were acquired after the fire event covering the entire burn and a 2 km buffer of unburned vegetation. We developed a methodology to address several specific objectives as follows: (1) to estimate aboveground biomass (AGB) from LiDAR data, (2) to quantify prefire and postfire biomass over the entire burned area, (3) to quantify the biomass consumed and the C released during the megafire, and (4) to assess estimation uncertainty.

## 2 Materials and Methods

### 2.1 Study Area

**Figure 1:**
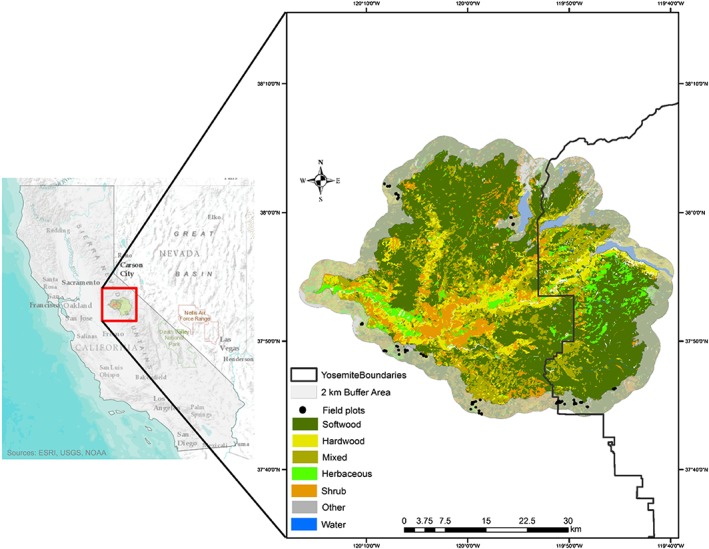
Study area comprising the footprint of the Rim fire, in the Sierra Nevada Mountains, California, USA.

The study site comprises the footprint of the Rim fire, in the Sierra Nevada Mountains, California, USA. This megafire started on 17 August 2013 and was not contained until 24 October 2013, burning more than 104,000 ha of the Stanislaus National Forest and Yosemite National Park (Figure 1). The area is topographically rough, with elevations ranging from 60 to 2400 m and slopes of up to 90%. The fire burned through a mosaic of vegetation types, which included low‐elevation grasslands, chaparral, and foothill‐oak woodland savanna habitat; mixed conifer‐broadleaf forests dominated by pines in the lower montane zone; and mixed conifer forests in higher elevation areas dominated by firs. A more detailed description of the study site can be found in *Casas et al*. [2016].

According to the burn severity map developed by the U.S. Forest Service Rapid Assessment of Vegetation Condition after Wildfire processing system [*Miller and Quayle*, 2015], the area experienced different levels of burn severity, with 35.8% in high burn severity, 25.4% in moderate severity, 30.9% in low severity, and 7.9% remained unburned.

### 2.2 Data Sets

#### 2.2.1 Reference Aboveground Biomass Data Sets

As part of the study, 71 circular plots of 0.09 ha (equivalent to the Landsat pixel resolution) were collected across a 2 km buffer zone around the fire perimeter. The plots were located following a stratified random sampling scheme using a Landsat‐based prefire vegetation map provided by the U.S. Forest Service. The strata were defined by vegetation type (softwood (conifers), hardwoods (broadleaf trees), and mixed forests) and diameter classes (12.7–25.2 cm, 25.2–50.6 cm, 50.6–76 cm, and >76 cm). Although the randomness of plot locations was slightly perturbed by accessibility constraints, the plots represented well the range of available biomass and were used to calibrate and validate the models based on the LiDAR data.

For each tree with diameter at breast height (DBH) greater than 10 cm, the species was recorded and the DBH tallied. The center of each plot was positioned using a differential GPS, with a horizontal accuracy after postprocessing better than 0.5 m. The spatial error in two plots exceeded 2 m, and these were discarded from further analysis. Additionally, fractional cover (FC) was estimated from the LiDAR data, and those plots having a FC less than 10% were also removed from the analysis. Thus, the final data set consisted in 65 field plots.

For each tree the total aboveground biomass (AGB) was computed using the National Biomass Estimator Library (NBEL) developed by the Forest Management Service Center (FMSC). The NBEL synthesizes published biomass equations for the U.S. and also unpublished allometric models developed by FMSC that are stored along with their associated metadata in a SQLite database [*Wang*, 2014]. The models used to estimate AGB were functions of DBH and tree height. The height was estimated from the measured DBH using the species level equations compiled by *Keyser and Dixon* [2012]. Table 1 summarizes the structural attributes of the sampled plots.

**Table 1:** Properties of the Plots Measured in the Field^a^

	DBH (cm)	Height (m)	AGB (Mg ha^−1^)	Stem Density (tree ha^−1^)
Maximum	142.5	53.6	645.4	1455.6
Minimum	10.0	4.9	24.21	22.2
Average	30.4	16.7	195.8	358.8
Standard deviation	19.9	8.5	143.1	263.7

a The plot size is 0.09 ha (Landsat OLI pixel size on the ground).

To extrapolate AGB values using Landsat OLI data, we used the LiDAR‐derived AGB as a reference. More than 500 pixels were randomly selected based on the histogram for the LiDAR‐derived AGB to ensure that the samples covered the full range of AGB values in the study area. Our sampling was limited to unburned and low‐severity areas. In this way, the effect of fire‐induced structural changes was eliminated from the sample.

#### 2.2.2 LiDAR Data and Processing

LiDAR data were collected on November 2013 by the National Center for Airborne Laser Mapping using an Optech Gemini Airborne Laser Terrain Mapper instrument that recorded up to four returns per pulse. These data covered the extent of the fire plus a 2 km buffer. The site was flown at a mean elevation of 2200 m above sea level with a maximum scan angle of ± 14° and a nominal 50% overlap between flight lines. The average point density was approximately 20 points m^−2^. The vendor provided the point cloud data set and a 1 m digital elevation model (DEM) that was used to normalize the height of each return.

We computed 16 variables related to canopy structure derived from the vertical structure of the forest measured by the LiDAR data, including height percentiles (H_25_, H_50_, H_75_, H_90_, and H_99_), the mean, standard deviation, kurtosis, skewness, and coefficient of variation for the height of the returns above 2 m. Biomass distribution within the canopy was estimated as follows: H_99_–H_50_, H_99_–H_25_, H_90_–H_50_, and H_90_–H_25_, as well as the canopy depth (maximum minus minimum vegetation height). Finally, the ratio of hits above 2 m to all returns was computed to represent canopy fractional cover [*Hopkinson and Chasmer*, 2009; *Morsdorf et al*., 2006] (Table S1 in the supporting information). These metrics have been shown to have strong relationships with the aboveground biomass [*Hudak et al*., 2012; *Næsset et al*., 2013].

In addition, we derived 19 metrics from the intensity of LiDAR returns after normalization to a standard range [*García et al*., 2010]. These metrics included intensity percentiles (I_25_, I_50_, I_75_, I_90_, and I_99_), the mean, kurtosis, range, skewness, coefficient of variation, and standard deviation, as well as accumulated intensity at H_25_, H_50_, H_75_, H_90_, and H_99_. The canopy FC was also estimated as the ratio of the canopy energy to the total energy [*García et al*., 2010; *Hopkinson and Chasmer*, 2009], as further detailed in Table S1 in the supporting information. We did not apply a correction factor to the intensity of the ground energy to account for the differences in reflectance between canopy and ground at the wavelength used by the LiDAR system, as suggested by other authors [*Lefsky et al*., 1999b; *Morsdorf et al*., 2006], because this factor is site dependent and it was not available for our study site. In addition, we computed the canopy reflection sum [*Means et al*., 1999] by summing the intensities of the canopy returns within the plot [*Hall et al*., 2006] and the density‐weighted canopy reflection sum [*García et al*., 2010], which accounts for variation in point density throughout the study site that results from topography or scan angle differences.

Following the approach described by *Muss et al*. [2011], we also constructed *pseudowaveforms* for each plot and derived six additional metrics related to the vegetation spatial distribution. These metrics included the height of the median energy and the height/median ratio, which are related to AGB [*Drake et al*., 2002; *Hyde et al*., 2005], the canopy height profile (CHP) describing canopy structure [*Lefsky et al*., 1999a], the mean canopy height, and the quadratic mean canopy height. In addition, we included the CHP variation coefficient and the area under the canopy waveform (AUCW) representing vertical heterogeneity and the amount of canopy material, respectively [*Bouvier et al*., 2015; *Muss et al*., 2011].

#### 2.2.3 Landsat OLI Data and Processing

We selected two orthorectified Landsat OLI reflectance images (path/row: 043/034) to estimate the biomass before and after the fire. The image dates were chosen based on the following two criteria: (1) minimize time gaps between the fire event and Landsat and LiDAR data acquisitions and (2) cloud and snow cover over the burned area must be zero. As a result, we chose the prefire image acquired on 30 July 2013 and the postfire image acquired on 18 October 2013. Although the postfire image was collected 6 days before the fire was fully contained, this was the only image that satisfied the above criteria. The next available image was acquired 1 year after the fire, and by then, cleaning and salvage logging operations had introduced structural changes that were undesirable for our purpose.

The imagery, downloaded from the U.S. Geological Survey Earth Explorer web site (http://earthexplorer.usgs.gov/; accessed: 21 August 2015), had been processed based on the Landsat Ecosystem Disturbance Adaptive Processing System (LEDAPS) atmospheric correction [*Masek et al*., 2006]. To ensure an appropriate coregistration between the different data sets used, the Landsat images were geocorrected by collecting ground control points (*n* > 25) using an intensity LiDAR image as reference and applying a linear transformation model, yielding an RMSE < 1 pixel.

Estimation of AGB and its dynamics from multispectral data relies on establishing relations between biomass and vegetation spectral response [*Foody and Mathur*, 2004; *Meng et al*., 2007; *Pflugmacher et al*., 2014], which is usually performed using spectral indices and/or transformations. We selected Landsat OLI reflectance bands 2 through 7 and derived four vegetation indices: the normalized difference vegetation index (NDVI) [*Tucker*, 1979], Normalized Difference Infrared Index (NDII) [*Hunt and Rock*, 1989], Enhanced Vegetation Index [*Huete et al*., 2002], and Visible Atmospherically Resistant Index [*Gitelson et al*., 2002]. We also used brightness, greenness, and wetness components of the tasseled cap transformation [*Kauth and Thomas*, 1976] using the OLI‐specific coefficients [*Baig et al*., 2014]. Tasseled cap components, particularly wetness, have been used to assess forest structure and forest change [*Cohen et al*., 1995; *Hansen et al*., 2001; *Pascual et al*., 2010]. From the brightness and greenness component we computed the tasseled cap angle and tasseled cap distance metrics [*Powell et al*., 2010] that are related to vegetation cover and vegetation composition and structure, respectively [*Pflugmacher et al*., 2014]. In Table S2 in the supporting information, we provide additional details.

Finally, for each band we calculated a set of isotropic texture metrics from the gray level co‐occurrence matrix for window sizes varying from 3 × 3 to 9 × 9 pixels. These metrics included the following: homogeneity, contrast, standard deviation, entropy, angular second moment, and correlation. They were previously found effective for biomass estimation in different forest ecosystems [*Kelsey and Neff*, 2014; *Lu*, 2005].

### 2.3 Ancillary Data

The DEM provided with the LiDAR data was resampled to 30 m using the average of all pixels included within the 30 m cell. Subsequently, the slope and aspect were computed for each pixel.

A Landsat‐based prefire vegetation map was provided by the U.S. Forest Service and reclassified into three vegetation type groups as follows: softwood, hardwoods, and mixed forest. These auxiliary layers were used in subsequent statistical analyses. A burn severity map derived from Landsat data, which was sensitive to different burn severity levels [*Key and Benson*, 2006], was also provided by the U.S. Forest Service, separating unburned areas from low, medium, and high severity burn levels.

### 2.4 AGB Estimation

In order to model AGB, we used a least squares support vector regression (SVR) approach (see supporting information for more details), using the LS‐SVMlab toolbox developed by *De Brabanter et al*. [2011] and implemented in MATLAB [*MATLAB*, R2010a]. Nonparametric regression techniques are attractive because they do not make explicit assumptions about data distributions and thus can model complex relationships between dependent and independent variables [*Zhao et al*., 2011]. We used a Gaussian radial basis function kernel, controlled by its bandwidth (*h*). The second parameter determining the estimation accuracy is the penalty parameter (*γ*), which represents a trade‐off between the model complexity and error on the training data. These two parameters were obtained by a grid search with tenfold cross validation.

A different support vector regression (SVR) model was developed for each of the remote sensing data sets we used. Given the large number of metrics derived from each data set, we used three methods for feature selection to reduce the processing time, increase model generalization performance, and help with the interpretation (see supporting information for more details) [*Weston et al*., 2000]. The model based on the metrics (features) derived from the LiDAR data was calibrated and validated using the field measurements described in section 2.2.1. However, to develop and validate the models based on the Landsat data (prefire and postfire biomass models), we used the LiDAR‐based AGB estimates, which allowed us to construct a much larger reference data set. Thus, for each date, we created a data set of 514 samples, with the features derived from the Landsat OLI image and the ancillary data. In both cases, approximately 70% of the samples were used for model training/calibration and the remaining samples were used for independent validation.

An initial evaluation of the LiDAR‐based postfire AGB estimates showed the presence of high AGB values within the high burn severity class. These areas had high H_50_ values, while the amount of canopy material (AUCW) was very low. Due to the strong correlation between H_50_ and AGB, this resulted in an overestimation of AGB in high burn severity areas. Therefore, we modeled the relationship between H_50_ and AUCW and derived a correction factor that was applied to the H_50_ metric to account for the changes in the distribution of canopy returns as a result of the fire. Subsequently, we recomputed AGB based on the weighted H_50_ (see supporting information for more details). Although the overestimation for H_50_ was most evident in high burn severity areas, we applied the AGB correction over the entire burn, including all severity levels. This was motivated by the fact that burn severity maps derived from Landsat data may not accurately capture the actual damage caused by the fire to understory and midstory vegetation in low and moderate severity areas [*Miller and Quayle*, 2015], especially if canopy cover is high. Hereinafter, the corrected AGB estimates are referred to as the “corrected LiDAR‐based AGB” or the AGB obtained with corrected LiDAR‐based data.

### 2.5 Biomass Consumption and Carbon Release Estimation

The amount of biomass consumed by the Rim fire was computed as the difference between the prefire AGB estimated from Landsat and the postfire AGB estimates derived from LiDAR and Landsat, as discussed above in section 2.4. Furthermore, *De Santis et al*. [2010] related the changes in different vegetation indices to combustion completeness, i.e., the fraction of biomass consumed by the fire. Therefore, in addition to the corrected LiDAR‐based postfire AGB, we also computed the difference between the prefire and postfire Landsat AGB estimates. In this way, we could evaluate the extent to which optical data alone can capture changes in biomass.

Finally, after computing the consumed biomass we estimated the released C by multiplying the biomass by a C fraction coefficient 0.5, as it is commonly done [*Marklund and Schoene*, 2006]. We reported the consumed biomass for the entire study area and also per burn severity levels.

### 2.6 Uncertainty Analysis

The performance of the SVR models was evaluated using the coefficient of determination (*R* ^2^), the adjusted coefficient of determination (*R* ^2^ adj), the root‐mean‐square error (RMSE), and the relative root‐mean‐square error (relRMSE) (Table S3 in the supporting information). The AGB extrapolation to the entire study area was based on a two‐step regression approach as follows: (i) first, field measurements were used to model AGB from LiDAR data and then (ii) the LiDAR‐based AGB so obtained was used to train the Landsat AGB model. The errors in these steps, denoted by RMSE_1_ and RMSE_2_, propagated to the final estimates. To calculate the overall error (variance) of the two‐step AGB estimator (i.e., *σ* ^2^_modeling_), we assumed the error of the individual steps to be independent, which leads to *σ* ^2^_modeling_ = RMSE_1_^2^ + RMSE_2_^2^. Other sources of error contributing to the overall uncertainty, such as remote sensing measurement error, sensor noise, field measurement error, and other sources (see supporting information), were not included in the uncertainty analysis and considered negligible compared to the error due to modeling. A comprehensive analysis of the error propagation and accounting for all error sources requires additional data not available for this study. The ground‐based AGB estimation error was considered negligible, as there was no uncertainty associated with the allometric equations used in NBEL.

We assessed the errors in the AGB and the biomass consumption estimates at a burn severity class level and combined these class‐wide errors into the overall, site‐wide quantities. This analysis accounted for spatial autocorrelation of the AGB error at the pixel scale using the techniques described by *McRoberts* [2006] and *Weisbin et al*. [2014] (details are provided in the supporting information). As the biomass consumption estimate is the difference between two correlated AGB estimates, its variance was estimated following *McRoberts et al*. [2014].

## 3 Results

### 3.1 AGB Estimates Using LiDAR Data

**Figure 2:**
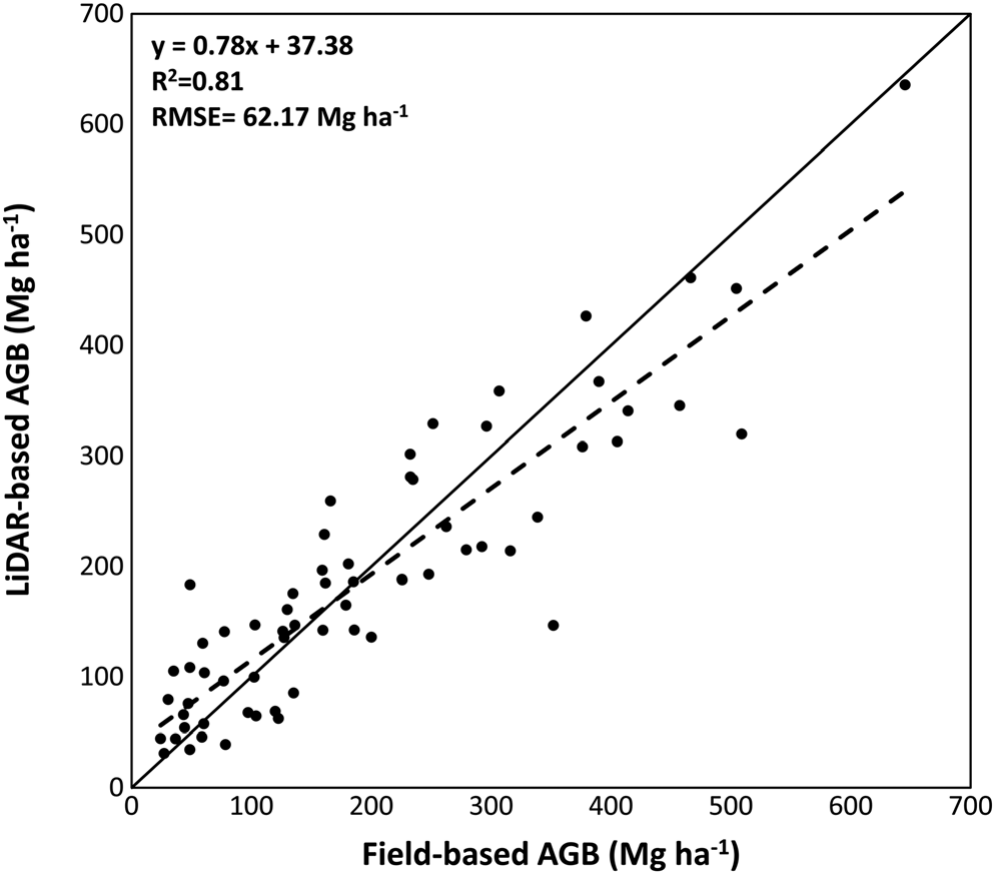
LiDAR‐based AGB estimates compared to field‐based AGB estimates. The solid line represents the 1:1 line.

Table 2 shows the results for the SVR model based on the metrics selected with one of the feature selection methods, the expert knowledge method. This model yielded the best results over the validation data and did not include correlated metrics as happened with the evolutionary algorithm. Therefore, this model was used to generate the LiDAR‐based AGB map for the study area. The results of the models built using the other two feature selection methods, the stepwise regression and the evolutionary algorithm, are given in Table S4 in the supporting information. Figure 2 shows the scatterplot of the LiDAR‐based versus field‐based AGB estimates.

**Table 2:** Accuracy of the SVR Model of AGB Based On Metrics Selected by Expert Knowledge^a^

Feature Selection	Selected Variables		*R* ^2^	*R* ^2^ Adj	RMSE (Mg ha^−1^)	relRMSE (%)
Expert knowledge	AUCW	H_50_	0.82	0.81	59.98	30.63
			0.82	0.79	67.18	34.31
			**0.81**	**0.80**	**62.17**	**31.76**

a For each accuracy measure, the first line corresponds to the calibration data set, the second line corresponds to the validation data set, and the third line (in bold) corresponds to the accuracy for the combined data sets.

**Figure 3:**
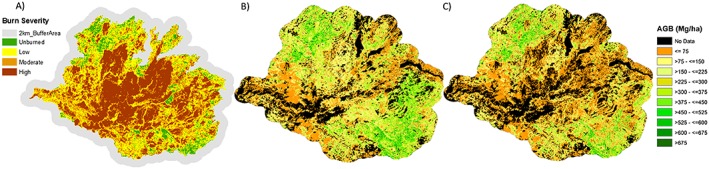
Spatial distribution of the postfire AGB estimated from LiDAR data: (a) burn severity map (added for comparison), (b) AGB using the original H_50_ metric, and (c) AGB using a corrected H_50_ metric (also referred to as the “corrected LiDAR AGB”).

The application of the SVR model using the H_50_ corrected based on the AUCW (section 2.4) showed significant differences compared to the original image. After correcting H_50_, the new AGB spatial distribution agreed better with the burn severity map (as illustrated in Figure 3), with an ~80% decrease in AGB in high burn severity areas and an ~50% reduction in uncertainty (Table 3). Between the two LiDAR‐based methods of AGB estimation, the AGB based on the corrected LiDAR data reflects more accurately the variation in the postfire AGB over the landscape (Figure 3). Table 3 also presents the mean AGB estimates and their uncertainties for different burn severity levels, vegetation types, and AGB estimation methods.

**Table 3:** AGB Mean ± Std Error (in Units of Mg ha^**−**1^) Within the Strata Defined by Burn Severity and Vegetation Type for Estimates Obtained Using LiDAR and Landsat OLI Data

Strata		LiDAR‐Based AGB	Corrected LiDAR‐Based AGB	Landsat‐Based Prefire AGB	Landsat‐Based Postfire AGB
Unburned	Coniferous	216.77 ± 0.64	216.77 ± 0.64	214.07 ± 1.49	209.39 ± 2.24
	Deciduous	74.81 ± 0.44	74.81 ± 0.44	81.64 ± 0.78	95.32 ± 1.55
	Mixed	147.56 ± 0.87	147.56 ± 0.87	115.74 ± 0.57	118.01 ± 0.70
Low severity	Coniferous	250.08 ± 0.45	183.81 ± 0.32	235.34 ± 0.79	232.69 ± 0.57
	Deciduous	101.42 ± 0.80	94.48 ± 0.42	96.77 ± 2.40	102.47 ± 0.93
	Mixed	193.71 ± 0.62	146.36 ± 0.54	162.81 ± 1.04	154.96 ± 0.96
Moderate severity	Coniferous	190.29 ± 0.54	90.65 ± 0.22	184.56 ± 1.09	122.58 ± 0.36
	Deciduous	76.74 ± 0.45	65.38 ± 0.28	82.86 ± 1.05	77.25 ± 0.62
	Mixed	144.28 ± 0.61	88.4 ± 0.35	136.58 ± 2.63	90.04 ± 0.57
High severity	Coniferous	151.05 ± 0.32	34.97 ± 0.14	172.13 ± 0.45	92.66 ± 0.28
	Deciduous	64.34 ± 0.36	30.60 ± 0.22	81.50 ± 1.84	92.53 ± 0.89
	Mixed	132.23 ± 0.44	33.89 ± 0.20	133.71 ± 0.55	90.46 ± 0.45

### 3.2 AGB Estimates Using Landsat OLI Data

**Figure 4:**
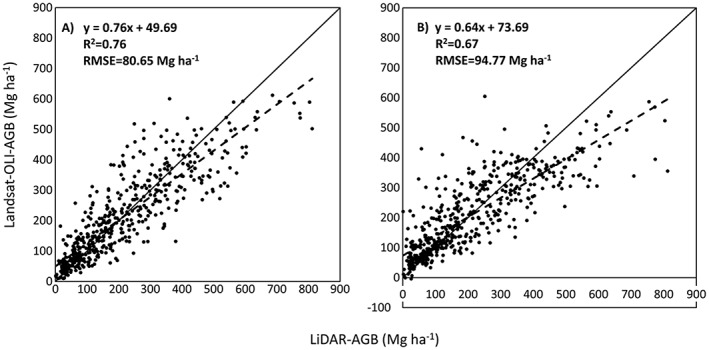
Scatterplots of LiDAR‐derived versus Landsat‐derived AGB for the (a) prefire and (b) postfire images.

Our first attempt to model AGB from Landsat data was based on a model that included texture that is the entropy of band 3 in a 9 × 9 window. This model yielded an *R* ^2^ of 0.73 and 0.61 and a relative RMSE of 46% and 56% for the calibration and the validation data, respectively. Nevertheless, when the AGB map was produced, it exhibited a clear pattern related to the entropy band rather than to the actual AGB distribution (Figure S4 in the supporting information). Therefore, we performed a second analysis that excluded texture measures to avoid this artifact. The final features selected to model AGB from Landsat were the OLI reflective bands 2 to 6, the NDII, and the elevation. Table 4 summarizes the performance of the prefire and postfire models. Figure 4 shows the scatterplots of the Landsat‐based versus LiDAR‐based AGB for each model.

**Table 4:** Features Selected From Landsat OLI for the SVR AGB Model (Left Most Column) and the Model Accuracy for Each Date^a^

Variables Selected	*R* ^2^	*R* ^2^ adj	RMSE (Mg ha^−1^)	relRMSE (%)
	*Prefire*			
B2‐B6, NDII, elevation	0.79	0.79	75.43	36.02
	0.72	0.71	87.82	41.94
	**0.76**	**0.76**	**80.65**	**38.52**
	*Postfire*			
	0.73	0.72	87.18	41.63
	0.6	0.58	105.01	50.15
	**0.67**	**0.67**	**94.77**	**45.26**

a For each accuracy measure and for each date, the first line corresponds to the calibration data set, the second line corresponds to the validation data set, and the third line (in bold) corresponds to the combined data set.

The combined uncertainty of the Landsat AGB estimates was computed using the RMSE of the models developed at each step (section 2.6) using the complete reference data set, i.e., the 65 plots for the LiDAR model and the 514 pixels for the Landsat model. This resulted in an overall relative RMSE of 55.3% and 49.9% for the postfire and prefire AGB, respectively. We also computed the uncertainties for the estimates of the mean AGB over areas with different burn severity levels and vegetation types. The uncertainty of the aggregated estimates was much smaller due to the large number of pixels and relatively weak spatial correlation (Table 3). However, the overall uncertainty of the postfire AGB estimates obtained with Landsat was double the uncertainty of corrected LiDAR‐based AGB, particularly in high severity regions where Landsat significantly overestimated LiDAR AGB by more than 150%.

### 3.3 Estimation of the Carbon Released by the Rim Fire

Table 5 presents the estimates for the biomass loss and C released to the atmosphere during the Rim fire using the three different postfire AGB estimates derived from the LiDAR and the Landsat data. In all cases, the AGB estimates were corrected for bias based on a linear regression between the observed and the estimated values (Figures 2 and 4). This process improves the dilution bias existing in the results but may introduce some random errors due to adjustments and changes introduced on all pixels, even with high estimation accuracy [*Xu et al*., 2016]. The estimates of the consumed biomass changed from 2.75 ± 0.21 Tg obtained using the original LiDAR‐derived AGB to 6.58 ± 0.03 Tg derived using the corrected LiDAR data. Applying a 50% biomass to carbon conversion factor, we obtained the estimates of the released C that ranged from 1.37 **±** 0.11 TgC to 3.29 **±** 0.02 TgC. For the Landsat‐based approach the consumed biomass estimate was 3.93 ± 0.17 Tg or 1.87 **±** 0.08 TgC.

**Table 5:** Biomass Consumed and C Released by the Rim Fire for the Entire Burned Area and per Burn Severity Level, Obtained Using Different Estimation Methods

			Burn Severity					
	Total Burned Area		Low		Moderate		High	
Method	Δ Biomass ± U (Tg)	ΔC ± U (TgC)	Δ Biomass ± U (Tg)	ΔC ± U (TgC)	Δ Biomass ± U (TgC)	ΔC ± U (TgC)	Δ Biomass ± U (TgC)	ΔC ± U (TgC)
Landsat_pre_‐Landsat_post_	3.93 **±** 0.17	1.96 **±** 0.09	0.90 **±** 0.17	0.45 **±** 0.09	1.30 **±** 0.01	0.65 **±** 0.01	1.73 **±** 0.02	0.86 **±** 0.01
Landsat_pre_‐LiDAR	2.75 ± 0.21	1.37 **±** 0.11	0.94 **±** 0.06	0.47 **±** 0.03	0.70 ± 0.2	0.35 **±** 0.01	1.11 ± 0.04	0.56 **±** 0.02
Landsat_pre_‐LiDAR_corrected_	6.58 ± 0.03	3.29 **±** 0.02	1.84 ± 0.03	0.92 **±** 0.02	1.67 ± 0.01	0.84 **±** 0.01	3.07 ± 0.01	1.53 **±** 0.01

**Figure 5:**
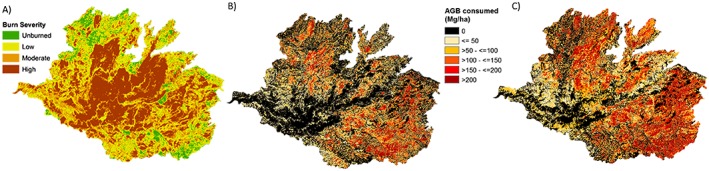
Spatial distribution of the burn severity and the aboveground biomass (AGB) consumed by the Rim fire obtained with two different estimation methods. (a) Burn severity map, (b) AGB_(Landsat;_ _prefire)_‐AGB_(Landsat;_ _postfire),_ and (c) AGB_(Landsat;_ _prefire)_‐AGB_(corrected_ _LiDAR;_ _postfire)_.

Figure 5 shows the spatial distribution of AGB difference between the Landsat prefire and the Landsat (Figure 5b) and LiDAR (Figure 5c) postfire estimates, representing the biomass consumption across the Rim fire domain. The difference between the LiDAR and Landsat data used for postfire AGB estimates can be readily observed in the significant underestimation by Landsat of the fire impact in terms of the biomass loss, particularly in the high severity areas. The AGB loss image derived from corrected LiDAR data shows the best visual agreement with the burn severity map (Figure 5a). Nonetheless, it can be seen that the match between the biomass difference map and the burn severity map is not perfect. One reason for this is that the burn severity map is only an approximate qualitative representation of the fire damage. Besides that, the burn severity map reflects a relative change in the overall vegetation spectral signal rather than an absolute measure of the biomass loss. Therefore, different burn severity levels could represent similar biomass losses depending on the prefire biomass levels and the extent and severity of forest disturbance, and the actual biomass loss can vary substantially within a burn severity class.

Furthermore, we quantified the biomass loss for each burn severity class and estimated the proportion of biomass consumed by the fire, i.e., the combustion completeness factor, for each severity level (Table 6). It can be seen from Table 6 that for all burn severity levels, both, Landsat and the uncorrected LiDAR data underestimated the CC values provided by *De Santis et al*. [2010] (last row in Table 6), while the corrected LiDAR data consistently overestimated those values.

**Table 6:** Combustion Completeness Factors Estimated by This Study Based On Different Data Sets and the Values Provided by *De Santis et al*. [2010] for a Mediterranean Conifer Forest in California

	Burn Severity		
Source	Low	Moderate	High
Landsat data	0.16	0.40	0.48
LiDAR data	0.16	0.22	0.31
Corrected LiDAR data	0.32	0.52	0.85
*De Santis et al*. [2010]	0.25	0.47	0.65

## 4 Discussion

### 4.1 LiDAR‐Based AGB Estimation

The LiDAR metrics that produced the best biomass model included 50th percentile canopy height (H_50_) and the area under canopy waveform (AUCW). The latter was also highly correlated with the canopy cover (*r* ^2^ = 0.90; *p* < 0.001), hence providing a description of the horizontal distribution of forest cover. LiDAR height metrics, such as percentiles or the mean of the height distribution, have been previously used as reasonable predictors of AGB, due to the relationship between AGB and canopy height. However, the inclusion of metrics such as fractional cover, which represents the horizontal variation of forest structure, is necessary to improve LiDAR‐based AGB estimation in heterogeneous forests [*García et al*., 2010; *Hall et al*., 2005; *Næsset and Gobakken*, 2008].

The estimation of AGB over burned areas with different severity levels was a critical step in our methodology. Since fires alter vegetation structure, ideally the model developed should capture the burn severity patterns and reflect these on the AGB estimates. A visual analysis of the LiDAR‐derived AGB map (Figure 3) shows that some areas of moderate and high burn severity still have large AGB values. Several reasons could explain this overestimation. On the one hand, pixels characterized by dead standing trees (snags) could present high H_50_ values despite much of their biomass having been significantly reduced compared to the prefire condition. On the other hand, the edge effect can significantly impact height values at 30 m resolution. These effects are a consequence of considering only returns with height > 2 m in the computation of H_50_. Although this is a common approach to compute LiDAR metrics [*García et al*., 2010; *Næsset and Gobakken*, 2008], which can be valid over undisturbed areas, the canopy structural changes caused by the fire affect the relationship between H_50_ and AGB resulting in an overestimation of AGB, thus making necessary a correction to account for the canopy reduction.

Although the model included the AUCW, which accounted for the amount of canopy material and partially compensated for this effect, the high correlation of H_50_ with AGB suggests a higher weight of this metric in the SVR model, thus biasing the estimates (see supporting information). Therefore, we corrected the bias in AGB values by modeling the relationship between H_50_ and AUCW. The lack of field data prevented us from validating this correction; however, the relation between tree height and crown size has been reported in other studies [*Mehtätalo et al*., 2014; *Popescu et al*., 2003]. It should be noted that the correction mainly affected pixels that experienced a large reduction in the canopy material, whereas pixels where trees retained leaves and/or branches and thus had high AUCW were barely affected by the correction.

### 4.2 Landsat‐Based AGB Estimation

Our final estimates of AGB are the result of a two‐step modeling approach, which yielded a final relative RMSE of approximately 50% for both, the prefire and the postfire estimates. Despite these high errors, our results are similar or even better than those reported in other studies that estimated AGB from Landsat data [*Frazier et al*., 2014; *Hall et al*., 2006; *Powell et al*., 2010].

In general, the relationship between Landsat spectral bands and biomass is extremely variable, depending on forest structure [*Hall et al*., 2006; *Jakubauskas and Price*, 1997]. There are examples showing the potential of Landsat reflectance for extrapolating forest structure over large regions [*Foody et al*., 2003]. However, such extrapolations can have large uncertainty at the pixel level, as shown in our study. In this work, several vegetation indices were employed for biomass modeling. The selection of NDII agrees with *Roberts et al*. [2004] and *Lu et al*. [2004] who found better relations between water sensitive indices and AGB as they exhibited greater sensitivity to large LAI values than NDVI.

The Landsat postfire model performed worse than the prefire model, probably due to illumination conditions in the fall, as well as phenological effects in the deciduous forest species, although these represented just 10% of the study area. Over the unburned areas, the average AGB estimate was similar to that of the LiDAR estimates (Table 3). However, over the burned areas, the postfire estimation of AGB had significant bias due to burn severity. Over high severity fire areas, Landsat estimates were three times greater than LiDAR over all forest types. Although in this work the postfire estimates were not compared with ground observations, the results show poor performance of Landsat spectral information compared to LiDAR to quantify the structural damage of the forests due to fire disturbance. LiDAR provides 3‐D information on tree structure that can be readily related to the damage on the live forest biomass; however, Landsat spectral reflectance may map areas of disturbance but have limited capability to capture the different degrees of vertical structural damage caused by fire [*Gajardo et al*., 2014; *Wulder et al*., 2009].

### 4.3 Estimation of Biomass Consumed and Carbon Released by the Rim Fire

The three data sets used to estimate the postfire AGB values yielded very different biomass consumption values. The use of the original LiDAR postfire AGB estimates produced the lowest biomass consumption. This is a result of the lack of sensitivity of Landsat data to high AGB values and the overestimation of AGB by LiDAR data over moderate and high burn severity areas, as described above. The estimate obtained from the corrected LiDAR data almost doubled that from Landsat. This difference can have multiple explanations. Over low and moderate burn severity areas Landsat data have limited ability to capture the changes in understory and midstory vegetation, particularly in high canopy cover forests, whereas LiDAR is more sensitive to these types of change. In addition, since burn severity can vary significantly within a pixel [*Miller and Quayle*, 2015], the presence of green vegetation within the pixel would cause an overestimation of AGB from Landsat. This would particularly affect low and moderate burn severity areas. Over high burn severity areas, our H_50_ correction approach reduced the bias in AGB estimation from LiDAR. Since no field measurements were made over these areas, no quantitative validation of the correction method was performed; nevertheless, our estimates from the corrected LiDAR data are in close agreement with initial estimates performed by the U.S. Forest Service [*U.S. Forest Service*, 2014].

Our estimate of the released C amounts to 12.06 ± 0.06 Tg of C02_e_, equivalent to the annual emissions of 2.57 million cars, as compared to the 11.35 Tg of C02_e_ reported by the U.S. Forest Service. Although our estimate is slightly higher, it should be born in mind that we excluded shrub vegetation from our analysis, and therefore, our values underestimate the total consumed biomass. Belowground biomass is usually not accounted for in biomass consumption computation; however, in some high severity areas of the Rim fire roots were also consumed, which could be a characteristic of megafires. Estimation of the consumption of this biomass component is challenging from both remote sensing and field observations, and further efforts are needed in this regard.

From the biomass consumption values obtained for each burn severity level, we estimated the proportion of biomass consumed by the fire (Table 6), i.e., the combustion completeness. The use of these CC values instead of a single value for the entire burned area or by vegetation type as it is commonly used would improve carbon release estimates based on the model proposed by *Seiler and Crutzen* [1980]. The CC estimates from the uncorrected LiDAR and Landsat data are lower than those reported by *De Santis et al*. [2010] for other Mediterranean conifer forest types in California. However, using the corrected LiDAR‐based AGB puts, our CC estimates in closer agreement, in most cases. Although *De Santis et al.* [2010] found good correlations between their CC estimates and temporal differences in vegetation indices, their results did not represent an actual estimate of biomass consumption, and therefore, it is difficult to assess the accuracy of their CC values. Differences between our results using Landsat data and the values by *De Santis et al*. [2010] can be explained, in part, by the fact that the postfire image used here was collected 1 week before the fire was fully contained. However, an image acquired after the complete extinction of the fire that also complied with our image selection criteria would have included forest changes due to postfire cleaning and salvage logging operations in the study area, which would have also affected the estimates.

## 5 Conclusions

Although airborne LiDAR is currently the most accurate remote sensing technology for biomass estimation, only few studies have evaluated its potential to characterize burned areas [*Kane et al*., 2014; *Wulder et al*., 2009], and to the best of our knowledge, it has not been used to estimate postfire AGB. We present a two‐phase methodology to extrapolate limited ground observations and airborne LiDAR‐based AGB estimates with Landsat multispectral imagery, both in space and time, to directly estimate the biomass consumed and the C released during the fire. In particular, we demonstrate how single‐date postfire airborne LiDAR data set can be integrated with Landsat imagery to overcome the limitations of spatial extent and frequency inherent in airborne LiDAR data to estimate the biomass by the fire.

The application of a correction factor to the LiDAR H_50_ metric over burned areas compensated for the systematic overestimation of the postfire AGB from LiDAR data and enabled the most accurate calculation of the biomass loss, compared to the original, uncorrected LiDAR or the Landsat data. It also improved the estimate of the combustion completeness factor for each burn severity level.

In addition, our methodology provided a formal uncertainty analysis and error propagation approach for calculating the uncertainty of the total emissions at different burn severity levels and sources of errors for future improvements. Suggested improvements would include addition of plots in burned areas to reduce biomass modeling uncertainty, optimization of plot size for the scale of the remote sensing data, and development of improved allometry for estimating the carbon stocks in standing burned trees.

## Supporting information



Supporting Information S1

Figure S1

Figure S2

Figure S3

Figure S4
