# Supplementary material for: Quantifying biomass consumption and carbon release from the California Rim fire by integrating airborne LiDAR and Landsat OLI data
Source: J Geophys Res Biogeosci. 2017 Feb 18;122(2):340–53. doi: 10.1002/2015JG003315 (PMC5367322; doi:10.1002/2015JG003315)
Supplement: Supplementary file 1 — Supporting Information S1 [file JGRG-122-340-s001.docx]

# Supporting information

Quantifying biomass consumption and carbon release from the California Rim Fire by integrating airborne LiDAR and Landsat OLI data

Mariano Garcia^1,2^, Sassan Saatchi^1^, Angeles Casas^3^, Alexander Koltunov^3^, Susan Ustin^3^, Carlos Ramirez^4^, Jorge Garcia-Gutierrez^5^, Heiko Balzter^2,6^

1. **LiDAR data processing**

The vertical and horizontal distribution of the vegetation was described by a set of metrics derived from the height and intensities distributions of the returns (table S1). The intensity of the returns is a function of many variables such as laser power, incidence angle, target reflectivity, illuminated area, atmospheric absorption and the range (sensor-target distance) [[*Coren and Sterzai*, 2006](#_ENREF_3); [*Korpela et al.*, 2010](#_ENREF_14)]; thus, it is necessary to calibrate these values in order to make them comparable between different scans, regions, or flights. Given the narrow scan angle used in our survey, the effect on the intensity values is negligible and can be ignored as shown in other studies [[*Coren and Sterzai*, 2006](#_ENREF_3); [*García et al.*, 2010](#_ENREF_5)]. Therefore, we applied a range normalization that eliminates the effect of path length variation on the intensity [[*García et al.*, 2010](#_ENREF_5)]:

| $I'=I*\frac{R^{2}}{R_{S}^{2}}$, | (S.1) |
| --- | --- |

where *I’* is the normalized intensity, *I* is the raw intensity value, *R* is the range (sensor-target distance) and *R*_s_ is the standard range (1000 m in this study).

Table S1 shows the intensity based metrics derived after normalizing the intensity data to a standard range.

Table S1: LiDAR metrics derived from the height and intensity distributions of the returns

| **Height metrics** | **Label** | **Intensity metrics** | **Label** | **Pseudo-waveform metrics** | **Label** |
| --- | --- | --- | --- | --- | --- |
| 25^th^ Percentile | H_25_ | 25^th^ Percentile intensity | I_25_ | Height of Median Energy | HOME |
| 50^th^ Percentile | H_50_ | 50^th^ Percentile intensity | I_50_ | Height to median ratio | HTRT |
| 75^th^ Percentile | H_75_ | 75^th^ Percentile intensity | I_75_ | Mean Canopy Height | MCH |
| 90^th^ Percentile | H_90_ | 90^th^ Percentile intensity | I_90_ | Quadratic Mean Canopy Height | QMCH |
| 99^th^ Percentile | H_99_ | 99^th^ Percentile intensity | I_99_ | Coefficient of Variation of the CHP | CVCHP |
| Mean height | Mean_h | Mean intensity | Mean_i | Area Under Canopy Waveform | AUCW |
| Standard deviation | Std_h | Standard deviation intensity | Std_i |  |  |
| Canopy depth | CD_h | Coefficient of Variation | CV_i |  |  |
| Kurtosis | Kurt_h | Range of intensities | Range_i |  |  |
| Skewness | Skew_h | Skewness | Skew_i |  |  |
| Coefficient of Variation | CV_h | Kurtosis | Kurt_i |  |  |
| 99th percentile - 50th percentile | H_99_- H_50_ | Canopy Cover | CC_i |  |  |
| 99th percentile – 25th percentile | H_99_- H_25_ | % of intensity accumulated at H_25_ | %Int_H_25_ |  |  |
| 90th percentile – 50th percentile | H_90_- H_50_ | % of intensity accumulated at H_50_ | %Int_ H_50_ |  |  |
| 90th percentile – 25th percentile | H_90_- H_25_ | % of intensity accumulated at H_75_ | %Int_ H_75_ |  |  |
| Canopy Cover | CC_h | % of intensity accumulated at H_90_ | %Int_ H_90_ |  |  |
|  |  | % of intensity accumulated at H_99_ | %Int_ H_99_ |  |  |
|  |  | Canopy Reflection Sum | CRS |  |  |
|  |  | Density Weighted Canopy Reflection Sum | DWCRS |  |  |

1. **Landsat OLI data processing**

Table S2 presents the formulation of the vegetation indices and variables computed from the Tasseled Cap components applied to the Landsat imagery.

Table S2: Spectral indices and band transformations applied to the Landsat OLI imagery

| **Spectral Index** | **Formulation** | **Parameters** |
| --- | --- | --- |
| Normalized Difference Vegetation Index -NDVI | $=\left( \frac{\rho_{NIR}-\rho_{R}}{\rho_{NIR}+\rho_{R}} \right)$ | ρ_R_: Reflectance in the red spectral region  ρ_NIR_: Reflectance in the near infrared spectral region  ρ_SWIR_: Reflectance in the shortwave infrared spectral region  G: Gain factor. Value = 2.5  C_1_& C_2_: Coefficients of the aerosol resistance term. Values = 6 & 7.5, respectively  L: Soil-adjustment factor. Value = 1  ρ_B_: Reflectance in the blue spectral region  ρ_G_: Reflectance in the green spectral region  TCG: Tasseled Cap Greenness  TCD: Tasseled Cap Brightness |
| Normalized Difference Infrared Index – NDII | $=\left( \frac{\rho_{NIR}-\rho_{SWIR}}{\rho_{NIR}+\rho_{SWIR}} \right)$ |  |
| Enhanced Vegetation Index – EVI | $=G\left( \frac{\rho_{NIR}-\rho_{R}}{\rho_{NIR}+{C_{1}*\rho}_{R}-{C_{2}*\rho}_{B}+L} \right)$ |  |
| Visible Atmospherically Resistant Index -VARI | $=\left( \frac{\rho_{G}-\rho_{R}}{\rho_{G}+\rho_{R}-\rho_{B}} \right)$ |  |
| Tasseled Cap Angle (TCA) | $= arctan(\frac{TCG}{TCB})$ |  |
| Tasseled Cap Distance (TCD) | $=\sqrt{{TCB}^{2}+{TCG}^{2}}$ |  |

1. **Feature Selection**

To broaden the information pool for SVR (Support Vector Regression) modeling, we initially derived a large number of candidate metrics (> 40) for each dataset and then applied a feature selection process. The feature selection process allowed us to understand the variables selected to model AGB, avoid overfitting problems, and improve processing times. For the LiDAR-AGB model, we evaluated three different feature selection methods. The first method was based on stepwise regression. This method initially includes the independent variable showing the highest R^2^ with the dependent variable. Additional variables are included into the model based on an F-test, under the assumption of normality of the variables. The p-value thresholds used to decide on addition/removal of the variables were set to 0.05 and 0.1, respectively. The second approach was based on an evolutionary (genetic algorithm) feature selection method developed by Huang and Wang [[2006](#_ENREF_9)]. This algorithm selects 100 initial different groups of features, i.e. the initial population. Then, it simulates the evolution of that population throughout a number of generations (in our case 100) where new offspring is generated by cross-over of the best individuals. To select the best individuals, the ability of each individual to predict AGB was evaluated with a 5-fold cross-validation. The group of features providing the best results in the final generation was finally selected to train the SVR. To reduce the effect of random influence common on genetic algorithms, it was run 10 times.

The last method was the expert knowledge approach. In order to select the most appropriate variables, we followed the approach proposed by Bouvier et al. [[2015](#_ENREF_2)] to develop models for the characterization of forest stand structure with high generalization potential. They develop predictive models of forest stand attributes based on four variables that capture the height and spatial distribution of forest stands, describing complementary 3D characteristics of the stand. Following this reasoning, we proposed a model based on two variables that were able to describe the 3D structural characteristics of the canopy, namely H_50_ and AUCW.

With regards to the Landsat-based models, we limited the feature selection method to the stepwise regression approach. The reasons for this were twofold: first, the evolutionary algorithm was extremely computationally demanding and thus impractical for the size of our database (>500 pixels). Second, the lack of *a priori* knowledge of what spectral and textural variables were related to the canopy properties of interest, as these variables are not directly related to forest biomass. The feature selection was conducted in two steps. In the first step, a stepwise regression was run using all Landsat metrics. In the second step, the correlation among the pre-selected variables was evaluated, and whenever they showed an inter-correlation > 0.7 we selected the one more highly correlated with the dependent variable.

1. **AGB modeling using Support Vector Regression**

We used a modeling approach based on Support Vector Machines (SVM), which is based on the principle of statistical learning theory and its foundations developed by Vapnik [[1995](#_ENREF_23)]. Although it was originally designed for binary classification purposes, it has been successfully applied for regression. Contrary to other approaches that are based on empirical risk minimization, that is, on the minimization of the training data error, SVM minimizes a structural risk by maximizing the margin between the separating hyperplane and the nearest training samples (termed support vectors) [[*Vapnik*, 1998](#_ENREF_24)]. In the case of Support Vector Regression (SVR), the SVM approach attempts to find the simplest regression model minimizing training data errors above a certain threshold [[*Smola and Scholkopf*, 2004](#_ENREF_20)]. A major drawback of SVM is its high computational burden involved in solving the quadratic programming problem required to find the optimal separating hyperplane. To overcome this drawback, Suykens and Wandewalle [[1999](#_ENREF_21)] proposed a least-squares approach, which solves a set of linear equations as an approximation for the quadratic programming problem.

SVM can be applied for nonlinear regression by mapping the input data into a higher-dimensional feature space, where the data will exhibit a linear pattern, using a kernel function. We used a Radial Basis Function (RBF) kernel which is controlled by the band-with of the kernel (*h*) and the regularization (penalty) parameter (*γ*). Appropriate selection of these parameters is critical to achieve good results while avoiding overfitting issues. These two parameters were obtained using a grid search approach with a ten-fold cross-validation.

It should be noted that the metrics (i.e. features) used as independent variables had different numeric ranges. Therefore, to avoid numerical difficulties and the bias resulting from features in greater numeric ranges dominating those in smaller numeric ranges [[*Hsu et al.*, 2009](#_ENREF_8)], we scaled the metrics to a range of [0,1] by considering the maximum and minimum values of each feature band.

| $X_{scaled}=\frac{X-X_{\min}}{X_{\max}-X_{\min}}$, | (S.2) |
| --- | --- |

where X and X_scaled_ respectively represent the original and scaled values for the feature at a pixel, and X_min_ and X_max_ are the minimum and maximum value for each feature band.

1. **Correction of LiDAR-based AGB estimates over burned areas**

The LiDAR-based AGB overestimation over high burn severity areas results from the fact that the LiDAR model used the median height (H_50_) and the AUCW as predictor variables. Despite the fire caused significant structural changes over these areas, many trees remained standing (snags), which produced high H_50_ values for LiDAR returns above the 2 m threshold used to compute the canopy metrics. The inclusion of the AUCW partially compensated for this effect since it is related to the fractional cover; however, H_50_ seemed to have a higher weight in the SVR than AUCW in the AGB estimation. Figure S1 shows an example of the canopy wave corresponding to two pixels of similar H_50_ over a high burn severity and an unburned area. The difference in the amount of canopy between them is evident. Thus, despite AUCW being 26.5 times higher for the unburned pixel, the AGB is only 1.6 times higher, which reflects the fact the AGB is mainly driven by H_50_ in our model and the inclusion of the AUCW compensated only partially for this effect.

*Insert Fig S1.*

To compensate for this effect we analyzed the relation between AUCW and H_50_ using more than1800 samples. Since taller trees generally have larger crowns, a positive relation between AUCW and H_50_ was expected. Figure S2 shows the scatter plot between the AUCW and H_50_ after binning the data to reduce the noise. We set the intercept to 0, since when there is no canopy material (AUCW = 0) then H_50_ should be 0.

*Insert Fig S2*

Based on this relation, we corrected the H_50_ values over the entire burned area. This correction mainly affected high burn severity areas having high H_50_ and low AUCW values, whereas for pixels that have high AUCW values the effect of the correction is negligible.

Figure S3 shows the H_50_, before and after the correction, and AUCW variables over the study area along with the burn severity map. It can be seen that the correction mainly affected high burn severity areas.

*Insert Fig S3.*

1. **Uncertainty analysis**

Table S3 shows the formulation of the variables used to assess the performance of the SVR models.

Table S3: Measures used for model performance evaluation

| **Accuracy measurement** | **Definition** | **Parameters** |
| --- | --- | --- |
| Coefficient of Determination (R^2^) | $1-\frac{SSE}{SST}$ | SSE: Sum of squares of residuals.  SST: Total sum of squares.  n: Number of observations.  p: Number of regression coefficients. |
| Adjusted Coefficient of Determination (R^2^-adj) | $1-\left( \frac{n-1}{n-p} \right)\frac{SSE}{SST}$ |  |
| Root Mean Square Error  (RMSE) | $\sqrt{\frac{\sum_{i=1}^{N} \left( P_{i}-O_{i} \right)^{2}}{n}}$ | O_i_: Observed value.  P_i_: Model-predicted value.  n. Number of observations. |
| Relative Root Mean Square Error (relRMSE) | $\frac{RMSE}{\mu_{AGB}}*100$ | μ_AGB_: Mean AGB value. |

In general, the overall uncertainty of remote sensing based AGB estimation should include various factors, such as field measurements, remote sensing measurements, image processing, and modeling approaches [[*McRoberts*, 2006](#_ENREF_16); [*Weisbin et al.*, 2014](#_ENREF_25)]. For example, [[*Weisbin et al.*, 2014](#_ENREF_25)] provide the following breakdown of the total variance for the AGB estimator at a pixel *u*:

| $\sigma_{AGB}^{2}\left( u \right)=\sigma_{RS data}^{2}\left( u \right)+\sigma_{modeling}^{2}\left( u \right)+\sigma_{field data}^{2}\left( u \right)+\sigma_{species variation}^{2}\left( u \right)+\sigma_{\Delta t}^{2}\left( u \right)$ | (S.3) |
| --- | --- |

where $\sigma_{RS data}^{2}$ (*u*) is the error of the remote sensing measurements (e.g., true canopy height vs. canopy height derived from LiDAR); $\sigma_{modeling}^{2}\left( u \right)$is the error in the SVR modeling; $\sigma_{field data}^{2}\left( u \right)$is the field measurement error (e.g., true vs. field-measured canopy height); $\sigma_{species variation}^{2}\left( u \right)$is the classification error in the vegetation map used to stratify models; and $\sigma_{\Delta t}^{2}\left( u \right)$ is the error due to the time gap between field and RS data acquisitions. In our study, we only estimated the errors associated with the two SVR modeling steps (sect. 2.6, main text), i.e. the $\sigma_{modeling}^{2}\left( u \right)$, since we did not have data to evaluate the accuracy of the RS, field, and vegetation map data.

The uncertainty of AGB estimation at the burn severity level was evaluated by integrating the pixel level errors over the regions of interest (ROI) and accounting for spatial autocorrelation of the errors as follows [[*McRoberts*, 2006](#_ENREF_16); [*Weisbin et al.*, 2014](#_ENREF_25)]:

| $\sigma_{AGB}^{2}\left( ROI \right)=\frac{1}{m^{2}}\sum_{i=1}^{m} \sum_{j=1}^{m} cov(\sigma_{i,}\sigma_{j})=\frac{1}{m^{2}}\left( \sum_{i=1}^{m} \sigma_{i}^{2}+2\sum_{i=1}^{m} \sum_{i<j}^{m} \rho(d)\sigma_{i}\sigma_{j} \right) ,$ | (S.4) |
| --- | --- |

where $\sigma_{AGB}^{2}\left( ROI \right)$ is the variance of the estimator for the mean AGB in the region of interest; *m* is the number of pixels in the ROI; ρ(d) is the spatial autocorrelation function of the distance d based on an exponential semi-variogram model; and $\sigma_{i}$ is the estimated standard error of AGB values at the i-th pixel, as discussed in the previous paragraph.

The change in biomass between times t_1_ and t_2_ was estimated as the difference between the corresponding AGB estimates:

| ${\Delta AGB}_{(t1-t2)}={AGB}_{t1}-{AGB}_{t2} ,$ | (S.5) |
| --- | --- |

Consequently, the variance of the estimator of mean biomass loss in an ROI (such as burn severity level) is given by the following equation:

| $\sigma_{\Delta AGB}^{2}\left( \text{ROI} \right)=\sigma_{AGB\left( t1 \right)}^{2}\left( \text{ROI} \right)+\sigma_{AGB\left( t2 \right)}^{2}\left( \text{ROI} \right)-2cov\left( {AGB}_{t1}, {AGB}_{t2} \right).$ | (S.6) |
| --- | --- |

The variances in the right-hand side of (6) were estimated by (4) and $cov\left( {AGB}_{t1}, {AGB}_{t2} \right)$ were estimated by the sample cross-time covariance of the AGB modeling error within the ROI (McRoberts, 2014):

| $\frac{1}{m^{2}}\sum_{i=1}^{m} \left( \sigma_{i}\left( t_{1} \right)- \bar{\sigma\left( t_{1} \right)} \right)\left( \sigma_{i}\left( t_{2} \right)- \bar{\sigma\left( t_{2} \right)} \right).$ | (S.7) |
| --- | --- |

The biomass loss estimate for the entire burn area, ${\Delta AGB}_{\text{Rim}}$ is a weighted sum of corresponding estimates for the burn severity classes: ${\Delta AGB}_{low}$ , ${\Delta AGB}_{mod}$ , and ${\Delta AGB}_{high}$, where weights are given by the relative contribution of these classes in the total biomass loss. Therefore, the standard error $\sigma_{\Delta AGB}\left( \text{Rim} \right)$ was computed as:

| $\sigma_{\Delta AGB}\left( \text{Rim} \right)= \frac{\sqrt{\left( {\Delta AGB}_{low}{\cdot\sigma}_{low} \right)^{2}+\left( {\Delta AGB}_{mod}\cdot\sigma_{mod} \right)^{2}+\left( {\Delta AGB}_{high}\cdot\sigma_{high} \right)^{2}}}{\vert{\Delta AGB}_{\text{Rim}}\vert},$ | (S.8) |
| --- | --- |

where $\sigma_{low}$ , $\sigma_{mod}$ , and $\sigma_{high}$ denote the standard errors of the burn severity level estimates of the biomass consumption.

1. **Results**
   1. **LiDAR based AGB estimates using different feature selection algorithms**

Table S4 shows the features selected by stepwise regression and by the evolutionary algorithms, as well as the accuracy of the SVR model using these features. The evolutionary algorithm selected AUCW, H_50,_ and mean height. The stepwise regression method selected H_50_ and two variables related to the canopy cover: DWCRS and the canopy cover estimated from return intensity.

The SVR model based on the variables selected by stepwise regression yielded the highest R^2^ and smallest RMSE for the calibration data and the lowest R^2^ and largest RMSE for the validation data (table S4). This reflects an overfitting of the model when these metrics were used, which limits its generalization potential. The SVR models derived using the variables selected with the evolutionary algorithm and expert knowledge gave almost identical results, as expected since they were based on nearly the same feature sets. In both cases the models demonstrated greater stability over the training and validation data that the stepwise approach, ensuring its applicability and generalization.

Table S4: Features selected and SVR-LiDAR AGB model accuracies obtained for each selected set of LiDAR variables

| **Feature Selection** | **Selected variables** | | | **R^2^** | **R^2^-adj** | **RMSE**  **(Mg ha^-1^)** | **relRMSE (%)** |
| --- | --- | --- | --- | --- | --- | --- | --- |
| Stepwise | H_50_ | DWCR | FC_i_ | 0.95 | 0.95 | 31.64 | 16.16 |
|  |  |  |  | 0.51 | 0.41 | 113.30 | 57.86 |
|  |  |  |  | **0.79** | **0.76** | **66.79** | **34.12** |
| Evolutionary | AUCW | H_50_ | Mean_H | 0.82 | 0.81 | 59.21 | 30.24 |
|  |  |  |  | 0.80 | 0.76 | 69.28 | 35.38 |
|  |  |  |  | **0.81** | **0.79** | **62.61** | **31.99** |

- 1. **Landsat based AGB estimates using texture metrics**

Our first attempt to model AGB from Landsat data was based on a model based on the brightness component of the Tasseled Cap transformation, the entropy of band 3 over a 9x9 window and the elevation data. This model showed a similar performance to the final model selected in our study. However, when the spatial distribution of AGB was mapped, a clear pattern of the texture was observed, as shown in figure S4. Although other studies have reported the suitability of texture metrics to estimate AGB from Landsat data [[*Kelsey and Neff*, 2014](#_ENREF_13); [*Lu*, 2005](#_ENREF_15)], the potential effect of these metrics on the spatial distribution of estimated AGB values should be considered.

*Insert Fig S4*

**List of figures**

Figure S1: Comparison of the canopy wave derived from a high burn severity pixel (dashed line) and an unburned pixel (solid line).

Fig S2. A linear model of the relationship between H50 and AUCW. The intercept is set to zero.

Fig S3. Spatial distribution of the explanatory variables used by the LiDAR-based SVR model. A) Burn severity map; B) Area under the canopy waveform (AUCW); C) Median height (H50) of vegetation returns; and D) Median height (H50) of vegetation returns corrected using linear regression on AUCW. After the correction, the median height distribution more closely corresponds to the burn severity map.

Fig S4. Effect of texture on the estimation of the biomass spatial distribution.
