# Supplementary figures and images for: Quantifying biomass consumption and carbon release from the California Rim fire by integrating airborne LiDAR and Landsat OLI data
Source: J Geophys Res Biogeosci. 2017 Feb 18;122(2):340–53. doi: 10.1002/2015JG003315 (PMC5367322; doi:10.1002/2015JG003315)

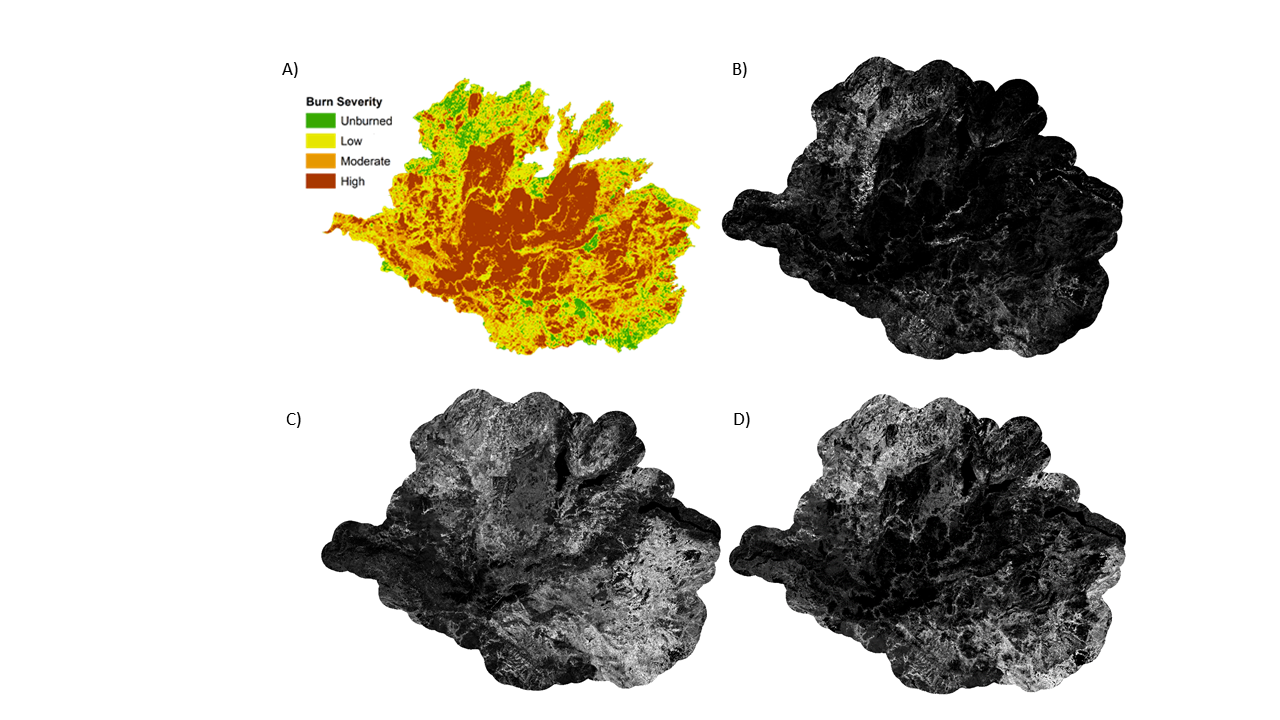

Supplement: Supplementary file 4 — Figure S3 [file JGRG-122-340-s004.tif]
